# Gene regulation analysis of patient-derived iPSCs and its CRISPR-corrected control provides a new tool for studying perturbations of *ELMOD3* c.512A>G mutation during the development of inherited hearing loss

**DOI:** 10.1371/journal.pone.0288640

**Published:** 2023-09-14

**Authors:** Xianlin Liu, Jie Wen, Xuezhong Liu, Anhai Chen, Sijun Li, Jing Liu, Jie Sun, Wei Gong, Xiaoming Kang, Zhili Feng, Chufeng He, Lingyun Mei, Jie Ling, Yong Feng

**Affiliations:** 1 Department of Otolaryngology, Xiangya Hospital, Central South University, Changsha, Hunan, China; 2 Key Laboratory of Otolaryngology Major Disease Research of Hunan Province, Changsha, Hunan, China; 3 Department of Otolaryngology Head and Neck Surgery, The Affiliated Changsha Central Hospital, Hengyang Medical School, University of South China, Changsha, Hunan, China; 4 Institute of Otolaryngology Head and Neck Surgery, University of South China, Changsha, Hunan, China; 5 Department of Otolaryngology, University of Miami Miller School of Medicine, Miami, FL, United States of America; 6 Department of Otolaryngology Head and Neck Surgery, The Eighth Affiliated Hospital, Sun Yat-sen University, Futian District, Shenzhen, China; 7 Medical Functional Experiment Center, School of Basic Medical Science, Central South University, Changsha, Hunan, China; University of Louisville, UNITED STATES

## Abstract

The *ELMOD3* gene is implicated in causing autosomal recessive/dominant non-syndromic hearing loss in humans. However, the etiology has yet to be completely elucidated. In this study, we generated a patient-derived iPSC line carrying *ELMOD3* c.512A>G mutation. In addition, the patient-derived iPSC line was corrected by CRISPR/Cas9 genome editing system. Then we applied RNA sequencing profiling to compare the patient-derived iPSC line with different controls, respectively (the healthy sibling-derived iPSCs and the CRISPR/Cas9 corrected iPSCs). Functional enrichment and PPI network analysis revealed that differentially expressed genes (DEGs) were enriched in the gene ontology, such as sensory epithelial development, intermediate filament cytoskeleton organization, and the regulation of ion transmembrane transport. Our current work provided a new tool for studying how disruption of ELMOD3 mechanistically drives hearing loss.

## Introduction

Human perception of sound depends on the refined structure of the cochlea, and the auditory sensory epithelium is the specialized region of the cochlea that transduces sound [1]. The organ of Corti located in the cochlea, contains one row of inner hair cells (IHCs) and three rows of outer hair cells (OHCs) [2]. The hair cells are lined with rows of actin-rich stereocilia arranged in a V-shaped staircase pattern on the apical surface of hair cells. The proper formation of this V-shaped structure is critical for facilitating proper hearing [3]. However, the mechanisms that drive proper hair cell function still need to be better understood. The studies of deafness genes improve our understanding of the development and function of hair cells.

Two pathogenic *ELMOD3* gene mutations have been found, leading to either autosomal recessive pre-speech hearing loss or autosomal dominant progressive hearing loss [4, 5]. Our group identified a heterozygous mutation in the *ELMOD3* gene (c.512A>G; p.His171Arg) that segregated with progressive non-syndromic hearing loss in a five-generation Chinese family in an autosomal dominant fashion [5]. *ELMOD3* gene belongs to the engulfment and cell motility family (ELMO family), which consists of six members defined by the presence of the ELMO domain [6]. ELMOD3 protein is an atypical GTP-activated protein (GAP) for the ARF family and plays roles in multiple cellular functions, including in primary cilia formation and traffic of cargoes from the Golgi to the primary cilium [7, 8]. In rat cochlea, Elmod3 is highly expressed in the stereocilia, kinocilia and cuticular plate in developing hair cells [4]. Moreover, we found in *Elmod3*^-/-^ mice, the absence of *Elmod3* resulted in abnormalities of cochlear hair cells in the manner of shortening and fusion of IHCs’ stereocilia and progressive degeneration of OHCs’ stereocilia [9]. However, little is known about how disruption of ELMOD3 mechanistically drives hearing loss.

**Fig 1:**
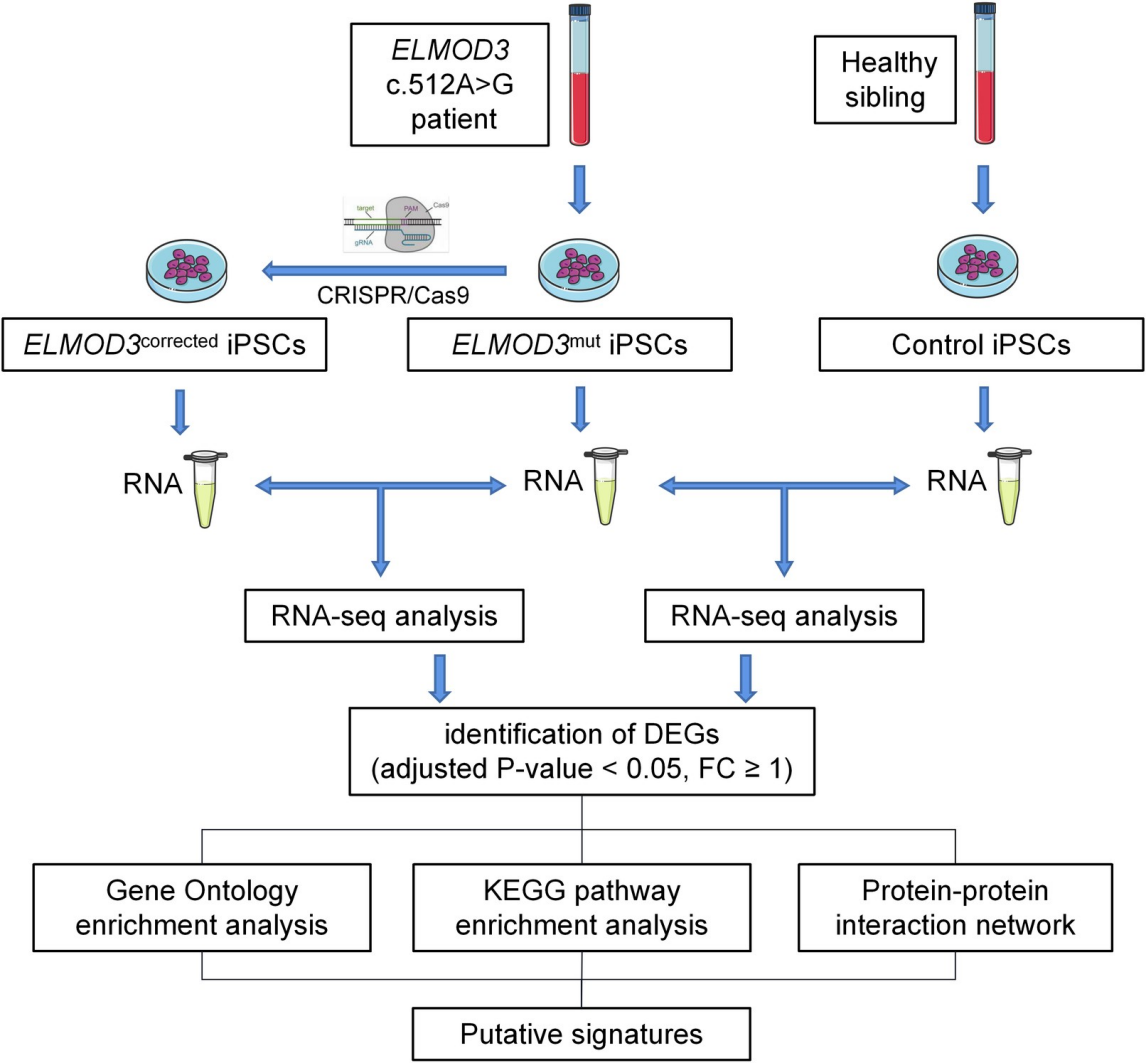
The overall workflow of this study.

Thus, using patient-specific human induced pluripotent stem cells (iPSCs) to generate an iPSC-based disease model may provide an approach to studying pathogenesis for hereditary hearing loss [10]. In this study, we generated a patient-specific iPSC line carrying the heterozygous *ELMOD3* c.512A>G mutation for the first time. Besides, we generated an iPSC line from a healthy sibling of the patient as control. The transcriptome analysis was conducted between the patient-specific iPSCs (*ELMOD3*^mut^) and the healthy sibling derived iPSCs (Control). Furthermore, we performed a gene correction of *ELMOD3* c.512A>G mutation iPSCs using CRISPR/Cas9 genome-editing technology, followed by gene profiling and transcriptome sequencing. We found several changes in differential gene expression by comparing the transcriptome of *ELMOD3*^mut^ iPSCs and its corrected isogenic control iPSCs (*ELMOD3*^corrected^). It was shown that the *ELMOD3* gene might be involved in biological processes, such as ion homeostasis and sensory epithelial development, etc. We also performed STRING and PPI analysis to predict the potential protein interaction network. Through these data, we could provide supportive evidence for the putative cellular function of *the ELMOD3* gene, thus yielding a model system to study how ELMOD3 functions in humans to facilitate proper hearing. The overall workflow of this study is shown in Fig 1.

## Materials and methods

### Ethics statement

The Ethics Committee of Xiangya Hospital Central South University (XHCSU) approved the protocol for this study, and signed informed consent was provided by every donor before sample collection. The laboratory research on the derivation and use of human iPSC lines was also approved by XHCSU following local regulations, and all animal experiments were conducted based on XHCSU ethical guidelines.

### Generation and culture of iPSCs

Ten ml of blood was collected from a hearing loss patient with *ELMOD3* c.512A>G mutation and a healthy family member in December 2019 [5]. The generation and culture of iPSCs were performed as described by Wen et al. [11]. The B lymphocytes were isolated from blood and immortalized by EBV. EBV-immortalized B lymphocytes were cultured in RPMI 1640 medium (Gibco, USA) with 10% fetal bovine serum (VISTECH, Australia) and 1 mm sodium pyruvate (Gibco, USA) at 37°C in 5% CO_2_. The expanded EBV-immortalized B lymphocytes were electroporated with two μg per vector of five episomal vectors (Addgene, USA), namely pCXLE-hUL, pCXLEhOCT3/4-shp53-F, pCXLE-hSK, pCXWB-EBNA1, and pCXLE-EGFP, for reprogramming into induced pluripotent stem cells (iPSCs). The Basic Nucleofector™ Kit for Primary Mammalian Epithelial Cells (Lonza, Switzerland) was used for electroporation. After electroporation, the cells were seeded in a 12-well plate and cultured in WiCell + VitC medium (DMEM/F12) (Gibco, USA). The medium should be replaced every 48h. From day 2 to day 12, 0.5 mM sodium butyrate (Sigma, USA) was added to the WiCell + VitC medium. Eight days after transduction, the cells were transferred to mouse embryonic fibroblast-coated 6-well plates at a density of 1.8×10^4^ cells per well. From day 12, the medium was changed to WiCell + VitC medium without sodium butyrate. Undifferentiated iPSC colonies were manually picked and transferred onto Matrigel-coated 24-well plates for further expansion.

### sgRNA design

The online tool CHOPCHOP (http://chopchop.cbu.uib.no/) was used to design sgRNA. The Lenti CRISPR v2 plasmid designed by Zhang’s laboratory was selected as the editing vector. The DNA fragments were ligated with the digested empty-loading plasmid using a DNA linking kit (Takara, Japan).

### Cell transfections

The four μg CRISPR/Cas9 targeting plasmids and 4μl ssODN (100μM) providing homologous recombination templates were transferred into 2.5×10^6^ patient-specific iPSCs by electrotransfection. Puromycin resistance genes were used as screening markers to obtain iPSCs successfully transfected. Then, these cells were transferred to culture plates at low density until sufficient clones appeared. Single clones were selected for Sanger sequencing.

### Alkaline phosphatase (AP) staining

Following the manufacturer’s instructions, the iPSCs were stained using Alkaline Phosphatase Kit (Beyotime, China). The photography was performed using a Nikon 300 inverted confocal microscope.

### Immunofluorescence staining

Cells were fixed with 4% paraformaldehyde for 20 min, permeabilized using 1% Triton X-100 (Sigma, USA) for 10 min, and blocked with 5% bovine serum albumin (BSA) for 1h. The above processes are carried out at room temperature. Following overnight incubation with primary antibodies in PBS solution with 5% BSA at 4°C, cells were washed and incubated with appropriate secondary antibodies for 1h at RT in the dark. DAPI (Beyotime, China) was used for nuclear counterstaining, and images were observed and photographed using an Olympus confocal microscope and camera.

### Embryoid body (EB) formation assay

For EB formation, iPSCs were harvested by Accutase (Gibco, USA), and then resuspended in the mTeSR medium (Stemcell Technologies, Canada) supplemented with 20% FBS. The cell suspension was transferred to ultra-low attachment six-well plates and cultured in KO-DMEM (Gibco, USA) supplemented with 20% Knockout Serum Replacement, 1% GlutaMax-I, and 1% nonessential amino acids. The medium should be replaced every 48h. After six days, The suspended spheroid embryoid body was collected and transferred to 6-well plates with gelatin-coated plates. Six days late, total RNA isolated from the embryoid body was reversely transcribed into cDNA for subsequent experiments. The expression of genes in different germ layers was examined by RT-PCR and agarose gel electrophoresis (AGE).

### Teratoma assay

The iPSCs (1 × 10^7^ cells) were harvested by Accutase (Gibco, USA) and injected into the hind limb muscles of 8-week-old male nude mice (Charles River, China). The teratomas were dissected 8 to 10 weeks post-transplantation, fixed with 4% PFA and embedded in paraffin. Tissue sections were stained with hematoxylin and eosin.

### Quantitative reverse transcription-polymerase chain reaction (qRT-PCR)

Total RNA was isolated from cells using the Trizol reagent (Sangon, China). One μg RNA was reverse transcribed using the PrimeScript™ II 1st Strand cDNA Synthesis Kit (Takara, Japan). The qPCR reactions were performed on Step One plus Real-Time PCR System (ABI) with a 2×SYBR Master Mix (Yeasen, China). The relative expression levels of target genes were calculated using the 2^- △△Ct^ method, and GAPDH served as the internal control.

### RNA sequencing

The total RNA of triple replicates from three samples (*ELMOD3*^mut^, *ELMOD3*^corrected^ and healthy control iPSCs) was extracted for further analysis. One μg of total RNA was isolated and used to generate RNA-seq libraries. The library preparations were sequenced on an Illumina Novaseq platform, generating 150 bp paired-end reads. The FPKM of each gene was obtained by quantitative analysis after filtering the raw data and reference genome alignment. Then we selected differentially expressed genes (DEGs) with the DESeq2 method (adjusted P-value < 0.05 and | log2 (fold-change) | > 1). To discern the implications of DEGs, we performed Gene Ontology (GO) and Kyoto Encyclopedia of Genes and Genomes (KEGG) pathway enrichment and protein-protein interactions (PPI) network analysis on the DEGs. The above analysis used the common online platforms and databases, including Metascape (http://metascape.org) [12], KEGG (http://www.genome.jp/kegg), the STRING (The Search Tool for the Retrieval of Interacting Genes/Proteins) database (https://string-db.org/), and Cytoscape (http://cytoscape.org/). The Molecular Complex Detection (MCODE) tool was used to extract functional modules.

## Results

### Generation of a human iPSC line from a patient carrying the heterozygous c.512A>G variant in the *ELMOD3* gene

In our previous study, a heterozygous *ELMOD3* c.512A>G (p.His171Arg) variant was identified as a causative variant in a five-generation Chinese family affected by late-onset and progressive autosomal dominant non-syndromic hearing loss (ADNSHL). The details about hearing loss history and auditory evaluation of the affected members were described previously by Li et al. [5]. Here, we generated two human iPSC lines by reprogramming the donated Epstein-Barr virus (EBV)-immortalized B lymphocytes of two family members (a 59-years-old male patient and his 51-years-old healthy female sibling respectively), henceforth named as *ELMOD3*^mut^ and healthy control. Detailed clinical characteristics of these siblings are shown in Table 1.

**Table 1:** Clinical characteristics of the patient and the healthy sibling.

	*ELMOD3* c.512A > G	Sex	Age	Onset Age	PTA threshold (dBHL)		Tinnitus	Vertigo
					Left	Right		
Patient	Yes	Male	59	37	89	83	No	No
Healthy sibling	No	Female	51	/	30	30	Yes	No

**Fig 2:**
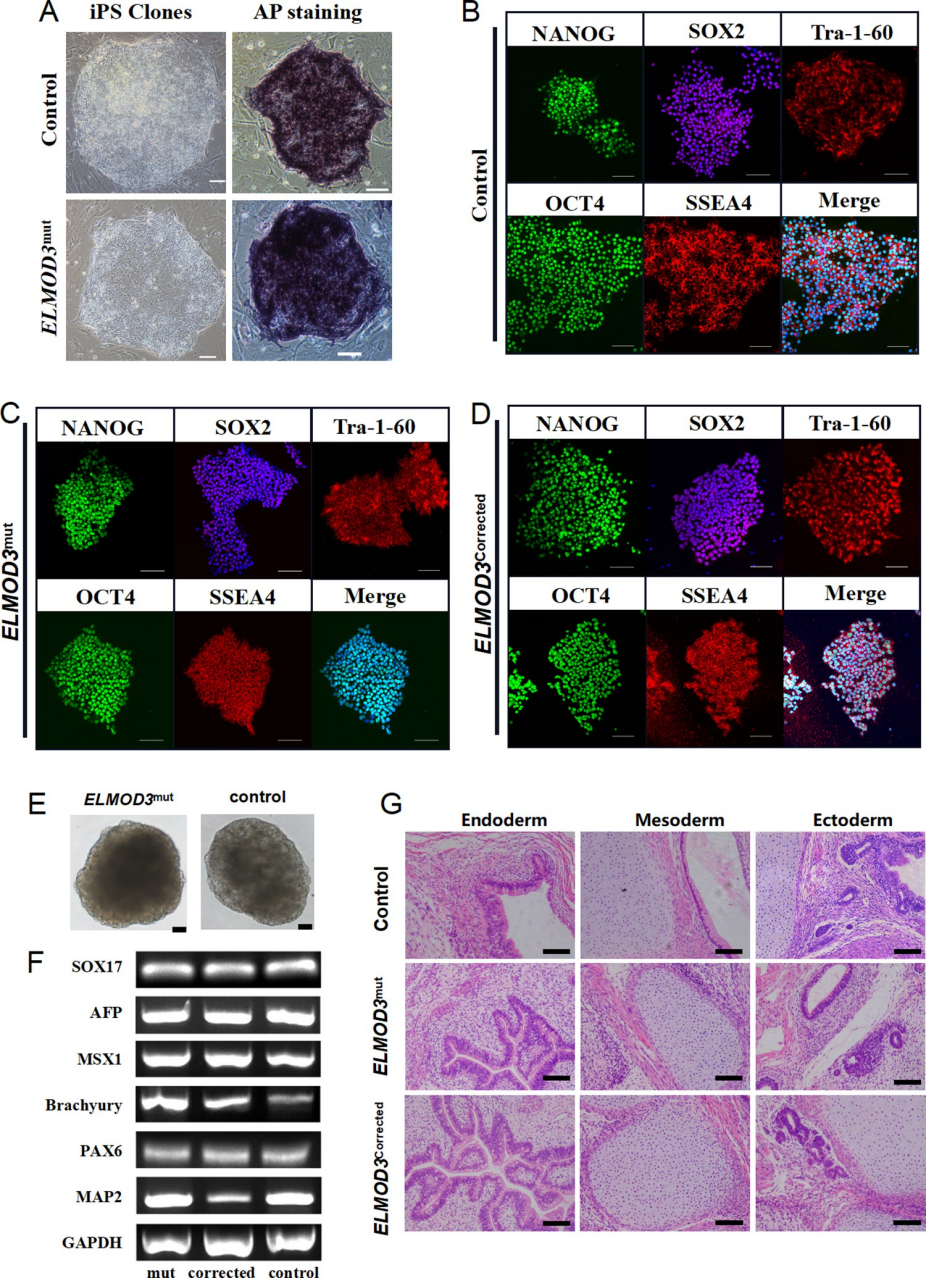
Generation and characterization of *ELMOD3*^mut^, control and *ELMOD3*^corrected^ iPSCs. (A) The *ELMOD3*^mut^ and control iPSC clones show typical embryonic stem cell-like and positive alkaline phosphatase (AP) staining. Bar, 100μM. (B-D) Immunofluorescence staining in all iPSC lines showed expression of pluripotency markers OCT4, NANOG, TRA-1-60, SOX2 and SSEA-4. Bar, 100μM. (E) The *ELMOD3*^mut^ and control iPSC line form embryoid body in vitro. Bar, 100μM. (F) The agarose gel electrophoresis of marker genes of three embryonic germ layers in all iPSC lines. (G) HE staining of teratomas generated from subcutaneous injection of all iPSC lines in NOD/SCID mice. Tumor sections represent differentiated structures as noted. Bar, 100μM.

Both the *ELMOD3*^mut^ and the healthy control iPSC lines exhibited a typical pluripotent stem cell-like morphology. The newly raised colonies after the reprogramming process appeared in blue when treated with an AP kit, showing that both iPSC lines presented AP activity and the potential of multi-differentiation (Fig 2A). The two studied clones were positive for NANOG, SOX2 and OCT-4 self-renewal markers, and also positive for SSEA-4 and Tra-1-60 pluripotency surface markers (Fig 2B and 2C). The two iPSCs can differentiate *in vitro* towards the three embryonic germ layers. RT-PCR analyses showed that the embryoid body expressed meaningful marker genes of endoderm, mesoderm and ectoderm (Fig 2E and 2F). Both iPSC lines were competent to differentiate into three germ layers in an *in vivo* teratoma assay (Fig 2G). To summarize, *ELMOD3*^mut^ and healthy control iPSC lines exhibited normal iPSC morphology, expressed pluripotency markers and differentiated into cells of three germ layers, which could be used in subsequent experiments.

We further observed the nuclear morphology and cytoskeleton of the *ELMOD3*^mut^ and healthy control iPSC lines. A high nuclear-cytoplasmic ratio was observed in both iPSC lines, and no significant difference in F-actin density between *ELMOD3*^mut^ and healthy control iPSC lines was observed (S1 Fig).

### Global changes in gene expression of the *ELMOD3*^mut^ iPSCs carrying the heterozygous c.512A>G mutation

**Fig 3:**
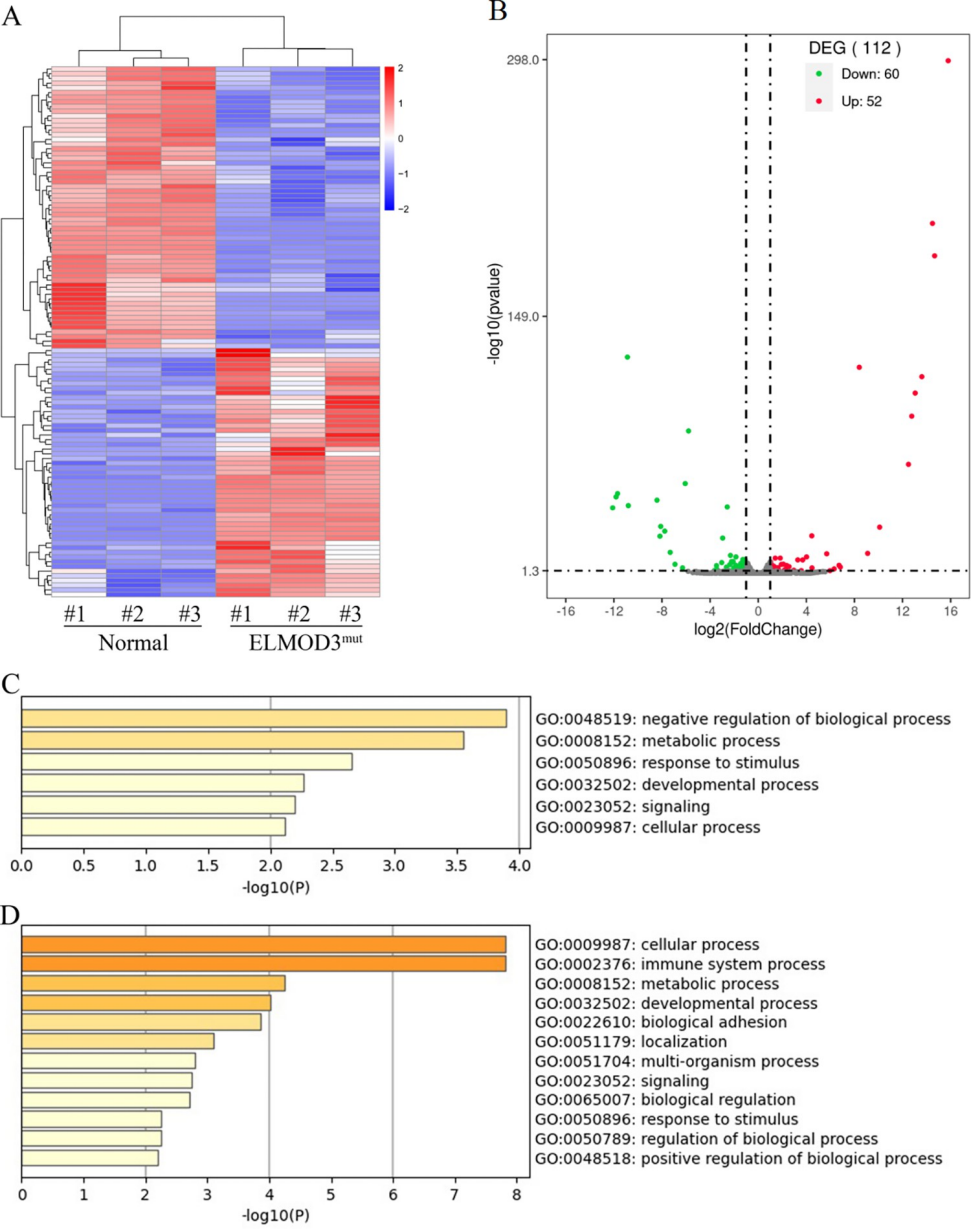
Differentially expressed genes between *ELMOD3*^mut^ and control iPSCs. (A) The heatmap showed a hierarchical clustering analysis of DEGs in *ELMOD3*^mut^ iPSCs. Red and blue indicate genes with high and low expression levels, respectively. (B) Volcano plot showing the expression change of each gene and their significance. Red dots represent the expression of genes in *ELMOD3*^mut^ iPSCs significantly up-regulated compared to normal control. Green dots represent the expression of genes in *ELMOD3*^mut^ iPSCs significantly down-regulated compared to normal control. (C-D) GO enrichment analysis of differently expressed genes in *ELMOD3*^mut^ iPSCs. GO terms enriched with up-regulated genes are shown in (C). GO terms enriched with down-regulated genes are shown in (D).

We applied RNA sequencing of the *ELMOD3*^mut^ and the healthy control iPSC lines, and a hierarchical cluster heatmap was generated (Fig 3A). The RNA sequencing statistics disclosed that there were 112 differentially expressed transcripts between *ELMOD3*^mut^ and control iPSC lines, of which 52 DEGs were up-regulated and 60 DEGs were down-regulated (Fig 3B, S1 and S2 Tables).

We utilized Gene Ontology (GO) analysis to investigate the biological processes associated with the DEGs. The up-regulated genes are mainly enriched in "negative regulation of biological process", "metabolic process", and "response to stimulus" GO terms (Fig 3C). In contrast, among the down-regulated genes caused by the *ELMOD3* c.512A>G mutation, "cellular process", "immune system process", and "metabolic process" are the most significantly enriched GO terms (Fig 3D). Interestingly, both up-regulated and down-regulated genes were enriched in identical biological processes, including the "metabolic process", "response to stimulus", and "developmental process".

However, there were not only *ELMOD3* gene mutations but also genomic differences (such as sex differences) among iPSCs derived from different individuals. Therefore we assume that global changes in gene expression between the above studied iPSC lines could not fully reflect the transcriptomic changes caused by *ELMOD3* c.512A>G mutation in humans.

### CRISPR/Cas9-mediated gene correction in the *ELMOD3*^mut^ patient-derived iPSCs

The clustered regularly interspaced short palindromic repeats (CRISPR)/CRISPR-associated protein 9 (Cas9) system is a powerful gene editing tool. Here, we used this system for gene repair in *ELMOD3* patient-derived iPSC line. The Small guide RNAs (sgRNA) were designed using the online tool CHOPCHOP (http://chopchop.cbu.uib.no/) and 16 suspicious off-target sequences were selected (see S3 Table for Off-targets sequences, S1A Fig). Sanger sequencing revealed that the *ELMOD3* c.512A>G mutation had been successfully corrected (S3B Fig). Then the 16 sequence regions were validated by PCR amplification and sequencing, showing that none of these 16 suspicious sites occurred (S3C Fig).

The generated iPSC line was named as *ELMOD3*^corrected^. Subsequently, the stemness-related gene expression and pluripotency in the *ELMOD3*^corrected^ iPSC were identified. Immunofluorescence staining indicated that the *ELMOD3*^corrected^ iPSC line could express the pluripotent stem cell marker NANOG, SOX2, Tra-1-60, OCT4, and SSEA4 (Fig 2D). We also tested its differentiation potential. The *ELMOD3*^corrected^ iPSC line can not only form embryos *in vitro*, but also grow in the subcutaneous muscles of NOD-SCID mice, showing that it has multiple differentiation potential (Fig 2G). In conclusion, the *ELMOD3*^corrected^ iPSC line has a multi-differentiation potential and was suitable for the following experiments.

### RNA-sequencing analysis of differentially expressed genes (DEGs) between *ELMOD3*^mut^ and *ELMOD3*^corrected^ iPSC lines

**Fig 4:**
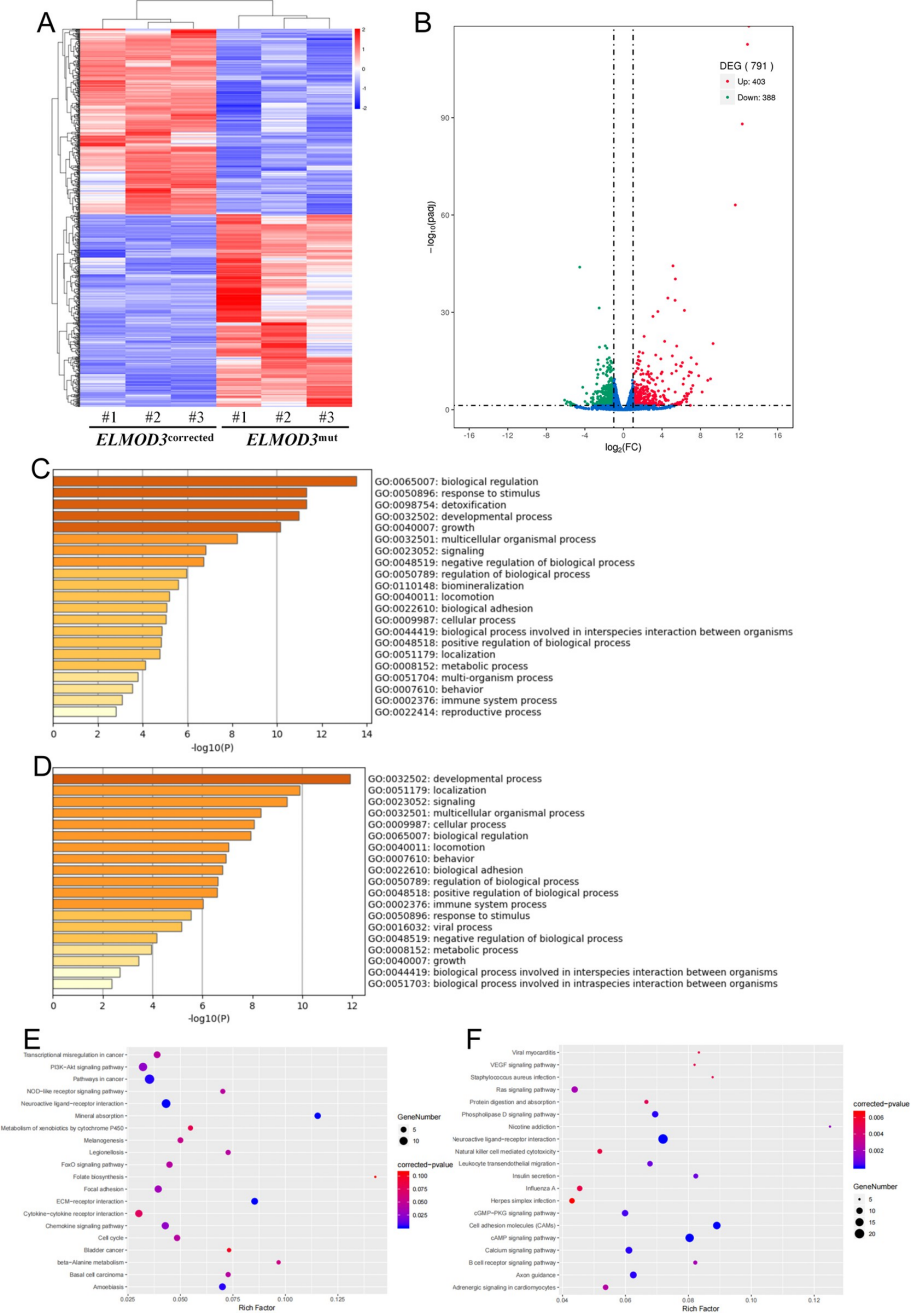
Differentially expressed genes between *ELMOD3*^mut^ and *ELMOD3*^corrected^ iPSCs. (A) The heatmap showed a hierarchical clustering analysis of DEGs between *ELMOD3*^mut^ and *ELMOD3*^corrected^ iPSCs. Red and blue indicate genes with high and low expression levels, respectively. (B) Volcano plot showing the expression change of each gene and their significance. Red dots represent the expression of genes in *ELMOD3*^mut^ iPSCs significantly up-regulated compared to Correction iPSCs. Green dots represent the expression of genes in *ELMOD3*^mut^ iPSCs significantly down-regulated compared to *ELMOD3*^corrected^ iPSCs. (C-D) GO enrichment analysis of differently expressed genes between *ELMOD3*^mut^ and *ELMOD3*^corrected^ iPSCs. GO terms enriched with up-regulated genes are shown in (C). GO terms enriched with down-regulated genes are shown in (D). (E-F) KEGG pathway enrichment analysis of DEGs specific for *ELMOD3*^mut^ iPSCs. Pathways enriched with up-regulated genes are shown in (E), while down-regulated genes are in (F).

A hierarchical cluster heatmap was generated to analyze the gene expression pattern (Fig 4A). A total of 789 DEGs were identified between *ELMOD3*^mut^ and *ELMOD3*^corrected^ iPSC lines, of which 403 were up-regulated and 386 were down-regulated (Fig 4B, S4 and S5 Tables). We found that *ELMOD3* c.512A>G mutation resulted in significantly down-regulated expression of genes encoding members of the Zinc-finger protein (ZNF) family.

Through GO analysis, 40 GO terms were significantly enriched (-log10 adjusted P-value > 2). We found that GO terms associated with "biological regulation", "response to stimulus", "developmental process", "growth", and "multicellular organismal process" were most significantly up-regulated (Fig 4C), while the down-regulated genes were mainly enriched in such as "developmental process", "localization", and "signaling" GO terms (Fig 4D).

Then, we performed KEGG pathway enrichment analysis for down-regulated and up-regulated genes. Focusing on significant enrichments, the up-regulated DEGs were involved mainly in pathways such as "mineral absorption", "neuroactive ligand-receptor interaction", "chemokine signaling pathway", and "PI3K-Akt signaling pathway" (Fig 4E). On the other hand, *ELMOD3*^mut^ iPSCs showed enrichment of down-regulated genes for pathways such as "neuroactive ligand-receptor interaction", "cAMP signaling pathway", "calcium signaling pathway", "axon guidance", and "cell adhesion molecules" (Fig 4F).

**Fig 5:**
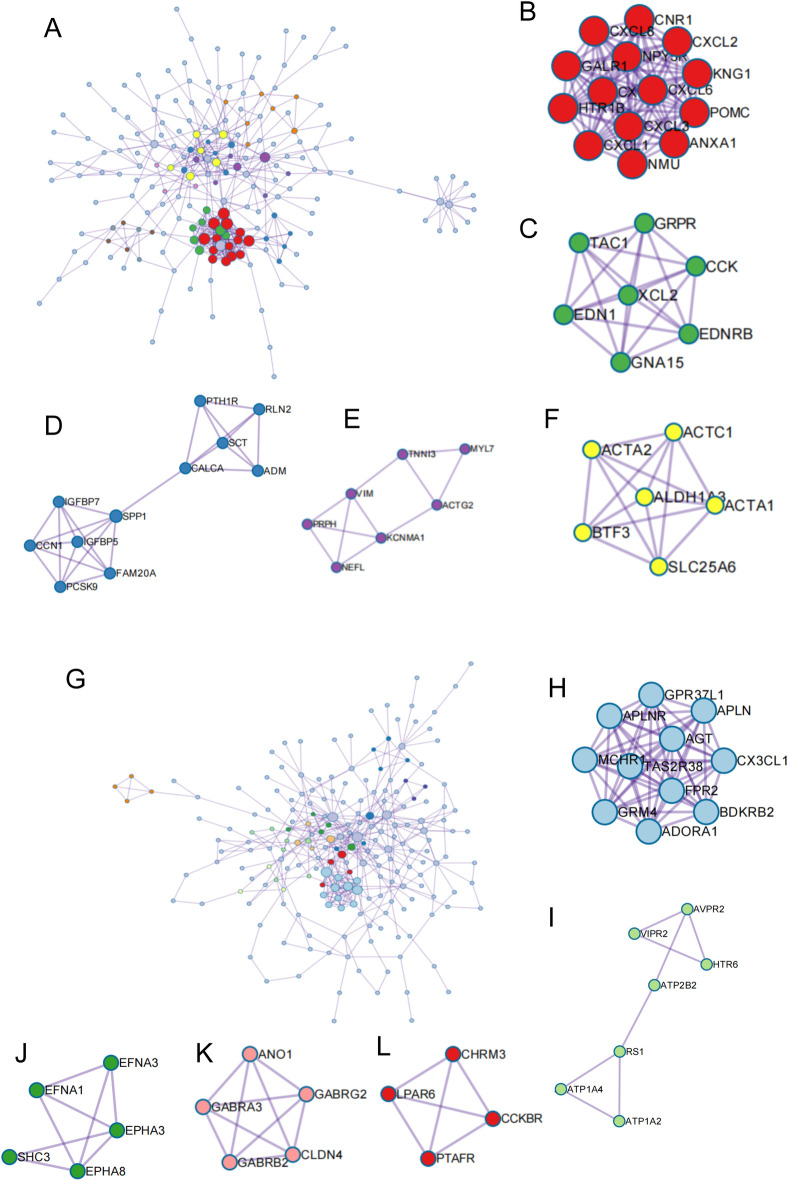
PPI network analysis of total DEGs in *ELMOD3*^mut^ and *ELMOD3*^corrected^ iPSCs. (A-F) PPI network of up-regulated DEGs and modules 1–5 identified from the whole PPI network. (A) the whole PPI network of up-regulated DEGs. (B) GPCR ligand binding. (C) GPCR downstream signalling. (D) Post-translational protein phosphorylation. (E) Ntermediate filament cytoskeleton organization. (F) mesenchyme migration. (G-L) PPI network of down-regulated DEGs and modules 1–5 identified from the whole PPI network. (G) the whole PPI network of down-regulated DEGs. (H) GPCR ligand binding. (I) cAMP signaling pathway. (J) Ephrin receptor signaling pathway. (K) Chloride transport. (L) Neuroactive ligand-receptor interaction.

Furthermore, PPI network analysis of predicted DEGs between *ELMOD3*^mut^ and *ELMOD3*^corrected^ iPSC lines was performed based on the STRING database. A total of 382 up-regulated genes (Fig 5A) and 361 down-regulated genes (Fig 5G) were mapped to the PPI network. The Molecular Complex Detection (MCODE) tool was used to extract functional modules. Among the up-regulated DEGs, genes related to "GPCR ligand binding", "GPCR downstream signaling", "post-translational protein phosphorylation", "intermediate filament cytoskeleton organization", and "mesenchyme migration" were significantly enriched (Fig 5B–5F). The down-regulated DEGs were most significantly enriched in "GPCR ligand binding", "cAMP signaling pathway", "ephrin receptor signaling pathway", "chloride transport", and "neuroactive ligand-receptor interaction" (Fig 5H–5L).

It is worth noting that, among the up-regulated DEGs in this study, one module included a total of 7 genes: *MYL7*, *TNNI3*, *VIM*, *PRPH*, *NEFL*, *KCNMA1*, and *ACTG2* was associated with "intermediate filament cytoskeleton organization" (Fig 5E), whereas another module consisted of a total of 6 genes: *ACTC1*, *ACTA1*, *ACTA2*, *ALDHA3*, *BTF3*, and *SLC25A6* were mainly enriched in "mesenchyme migration" (Fig 5F). Amongst these DEGs, four genes were tested in a qRT-PCR assay and the expression of tested genes was significantly up-regulated in *ELMOD3*^mut^ iPSCs, which was consistent with the RNA-seq results (S3A and S3B Fig). These findings echo our earlier research that defects in the *Elmod3* gene caused the inner ear hair cell stereocilia structural abnormalities *in mice*.

**Fig 6:**
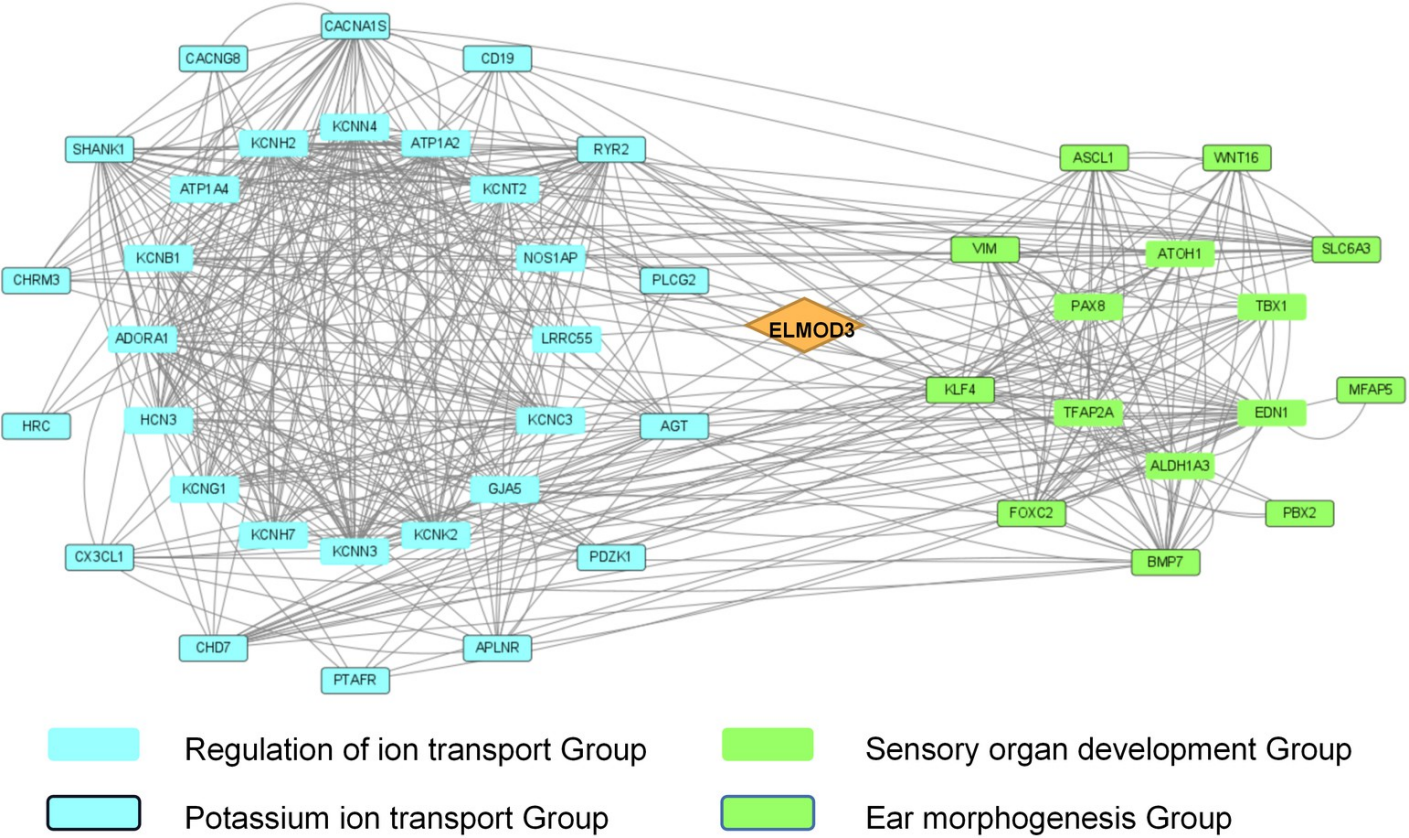
Integrated analysis of specific gene sets in DEGs of *ELMOD3*^mut^ and *ELMOD3*^corrected^ iPSCs. The diamond represents *ELMOD3*. The blue square represents the genes enriched in the GO term regulation of ion transport, and the blue square with the frame indicates genes related to potassium ion transport. The green square represents the genes enriched in the GO term sensory organ development, and the green square with the frame indicates genes related to ear morphogenesis.

Moreover, we also found DEGs related to neural development and ion transport, especially potassium ion transport. There were 26 down-regulated genes linked to "regulation of ion transmembrane transport", including *LRRC55*, *CACNG8*, *NOS1AP*, *KCNH2*, *GJA5*, etc., of which 16 genes linked to "potassium ion transport" including *KCNB1*, *KCNC3*, *KCNG1*, *KCNH2*, *KCNK2*, *KCNN3*, and *KCNN4* (Fig 6, left). In the *ELMOD3*^mut^ iPSC line, genes related to "sensory organ development", especially "ear morphogenesis" were significantly up-regulated, including *TBX1*, *ATOH1* and *ALDH1A3* etc. (Fig 6, right). We verified the expression levels of several of these genes by qRT-PCR, and the result was also consistent with RNA-seq results (S3C–S3F Fig).

## Discussion

Hearing loss (HL) is one of the world’s most common sensory deficits with exceptionally high genetic heterogeneity [13]. 50% to 60% of hearing loss in babies is due to genetic causes [14]. Different mutations at one causative gene could result in different patterns of inheritance and various phenotypes. For example, *ELMOD3* mutation (c.794 T>C; p.Leu265Ser) is responsible for autosomal recessive non-syndromic HL and another *ELMOD3* mutation (c.512A>G; p.His171Arg) causes non-syndromic progressive HL in an autosomal dominant fashion [4, 5]. Deafness genes, such as *GJB2* and *TMC1*, show similar clinical manifestations [15–18]. In this case, it is necessary to carry out a series of animal and cell studies on different mutation sites for these genes.

The ELMOD3 protein belongs to the engulfment and cell motility family (ELMO family), which has the same ELMO domain. The human ELMO protein family consists of six members, which could be further divided into two subgroups, ELMOs and ELMODs, based on protein size and domain architecture [6]. The ELMOD proteins share GAP activity and a conserved ELMO domain, a ~160 residue domain also present in human ELMO proteins [7, 19]. The c.512A>G mutation in *the ELMOD3* gene was found in amino acid 171, substituting a highly conserved histidine residue with arginine [5]. In a previous study, we confirmed that *Elmod3*^-/-^ mice exhibited progressive hearing loss and hair cells stereocilia morphology anomalies [9]. Whereas the mechanisms underlying stereocilia morphology anomalies are still not fully understood. There are many differences in phenotype between animal and human models due to differences in genetic background, organ development timelines, and underlying regulatory mechanisms between species [20, 21]. Therefore, in this study we chose to generate patient-specific human iPSCs carrying c.512A>G mutation, which could be used as an iPSC-based disease model for pursuing how disruption of ELMOD3 causes hearing loss.

Since 1998, when Thomson and his colleagues reported the first human embryonic stem cell lines [22], iPSCs have led to a better understanding of the mechanisms that mediate normal and abnormal early human development. Personalized iPSC lines can be derived, enabling studies of the molecular pathogenesis of inherited diseases [23]. Given the inner ear’s complex cellular and molecular structure, and all cell types within the inner ear are present in small amounts, iPSC-based modeling of hearing and hearing loss promises to revolutionize scientific approaches to hearing loss [24]. In the past few years, iPSCs have begun to play an important role in illuminating the details of human inner ear development, particularly the formation of functional hair cells [25, 26].

In the current work, we successfully reprogrammed B lymphocytes from a hearing loss patient with *ELMOD3* c.512A>G mutation into iPSCs, based on the Yamanaka method [27]. The iPSCs cell model established in this study is not only an effective tool to study how disruption of ELMOD3 mechanistically drives hearing loss, but also provides a potential tool to carry out gene therapy research in the future. To our knowledge, this is the first documented disease model of induced pluripotent stem cells from deafness patients caused by an *ELMOD3* gene mutation.

When new iPSC lines are developed, it is essential to perform exhaustive characterization of the new lines [28]. We have carried out a panel of assays, including morphological observation, AP activity detection, pluripotency marker expression, and teratoma formation potential. The results showed that *ELMOD3* mutant iPSCs exhibited similar pluripotency properties as control iPSCs. A comprehensive transcriptome profiling of iPSCs from patients with the heterozygous *ELMOD3* c.512A>G mutation was performed using next-generation RNA-Sequencing. Compared to control iPSCs, transcriptome analysis of *ELMOD3*^mut^ iPSCs showed that there were 112 DEGs significantly altered. However, the DEGs in the above transcriptomics analysis could be biased due to different gender and other genomic differences between this patient and his healthy sister. For example, the top 5 up-regulated genes of *ELMOD3*^mut^ iPSCs were located on the Y chromosome (*RPS4Y1*, *KDM5D*, *USP9Y*, *NLGN4Y* and *EIF1AY*).

In order to exclude such differences in gene expression profiles between different individuals, we also generate a corrected isogenic control iPSCs (*ELMOD3*^corrected^) using CRISPR/Cas9 technology, which is based on the single-guide RNA (sgRNA) encoding DNA-binding specificity and combined with a single nuclease (Cas9) [29]. Sanger sequencing revealed that the *ELMOD3* c.512A>G mutation was successfully corrected, and no off-targeting had occurred. After genetic correction with CRISPR/Cas9, the *ELMOD3*^corrected^ iPSCs retained the multiple differentiation potential. Compared with the control iPSCs, several genes located on the Y chromosome whose expression was significantly up-regulated in the *ELMOD3*^corrected^ iPSCs (S6 Table). Of the 35 up-regulated genes shared between *ELMOD3*^mut^ group (*ELMOD3*^mut^ vs control) and *ELMOD3*^corrected^ group (*ELMOD3*^corrected^ vs control), 11 were located on the Y chromosome. This indicates that gender factors should be considered in transcriptome analysis if different gender samples are used.

Transcriptome comparison between *ELMOD3*^mut^ and *ELMOD3*^corrected^ iPSC lines revealed more exciting findings. The transcriptional landscapes of the above two studies are quite different, while we still find some features in common, such as two sets of DEGs were both enriched in "response to stimulus" and "developmental process". It should be noted that compared to *ELMOD3*^corrected^ iPSCs, the *ELMOD3* c.512A>G mutations led to the up-regulation of genes associated with the biological process “sensory epithelial development”. Moreover, previous studies showed that ELMOD3 was expressed in the stereocilia of hair cells in rat and mouse models [4, 9], and defects in the ELMOD3 gene lead to morphological abnormalities of the hair cells’ stereocilia in mice [9]. Deletion of Elmod1 *in vitro* results in the decreased ability of cells to form primary cilia, and the loss of a subset of proteins from cilia [8]. ELMOD3 interacts with Rab1A and Flotillin2 to regulate lumen formation via vesicle trafficking [30]. The current work found a series of DEGs related to sensory epithelial development and cytoskeleton organization through functional enrichment and PPI network analysis, including *ATOH1*, *VIM*, *PRPH* and NEFL, which may be involved in the pathogenesis of *ELMOD3* causing deafness.

*ATOH1* (*Atonal BHLH Transcription Factor 1)* plays a crucial role in hair cell development and maturation [31]. *Atoh*1^-/-^ mice exhibited severe hearing loss and vestibular dysfunction, with complete loss of cochlear hair cells and vestibular hair cells [32]. In E15.5-E17.5, Atoh1 depletion resulted in rapid hair cell death, but the removal of Atoh1 after this period did not affect the number of new hair cells [33]. Overexpression of Atoh1 induces hair cell regeneration in the adult cochlea [34]. Our study showed that *ELMOD3* mutation resulted in increased expression of *ATOH1*. This may be the compensation for the effects of *ELMOD3* mutations. However, with the cochlea’s maturation, cochlear cells’ ability to respond to Atoh1 will be gradually weakened [35]. Whether ELMOD3 and ATOH1 interacted with each other is worth exploring in future studies. Furthermore, previous studies on the mechanism of *ELMOD3* induced deafness mainly focused on abnormal stereocilia morphology. Loi et al. found that *ELMOD3-SH2D6* gene fusion leads to autism spectrum disorders [36], which suggests that the ELMOD3 gene has more functions yet to be discovered.

We also found that several DEGs linked to the biological process “potassium ion transport” were significantly down-regulated. Hereditary progressive hearing loss is closely related to decreased cell surface expression and impaired potassium channel conductance in outer hair cells [37]. The human genome contains roughly 70 K^+^ channel-encoding genes [38]. Many of these genes play essential roles in auditory processes, such as *KCNQ4* [39], *KCNQ1* [40], *KCNMA1* [41] and so on. They are expressed in different parts of the cochlea and play different roles. In both studied transcriptome analyses, *LRRC55* expression was significantly down-regulated. *LRRC55* belongs to large conductance K^+^ channels, termed BK channels, which play an essential role in cell excitability and maintenance of K^+^ homeostasis [42]. BK channel expression has been identified in the cell bodies of SGN and hair cells [41, 43]. Compared to *ELMOD3*^corrected^ iPSCs, there were 26 down-regulated genes linked to "regulation of ion transmembrane transport" and 16 genes linked to "potassium ion transport" were down-regulated in the *ELMOD3*^mut^ iPSC line. These results suggest that ELMOD3 might be involved in regulating cochlear K^+^ homeostasis.

In summary, we generated a patient-specific iPSC line carrying *ELMOD3* c.512A>G mutation and a healthy control iPSC line for the first time. Subsequently, the patient-specific iPSCs were corrected by CRISPR/Cas9. In addition, we applied transcriptome profiling to compare these iPSC lines. In-depth analysis of high throughput RNA sequencing data between the patient-specific iPSCs and the corrected iPSCs, we attempt to know more about how disruption of ELMOD3 mechanistically drives hearing loss. Although we should acknowledge that this study is based only on transcript levels, our current work provided a new tool for studying the cellular function of ELMOD3 and gave hints about biological processes which may be related to ELMOD3. ELMOD3 may affect the process of sensory epithelial development and the regulation of ion transmembrane transport via some other proteins. Thus, more extensive studies and awareness of these associations are needed. Also, if inner-ear-like tissue could be derived from these iPSC lines in the next step, coupled with transcriptome and proteome profiling could be conducted at that stage, we could further extend our understanding of the molecular etiology of ELMOD3 causing hearing loss.

## Supporting information

S1 Fig Immunofluorescence staining of the nuclear morphology and cytoskeleton of all iPSC lines. Bar, 100μM.
(TIF)

S2 Fig Establishment of correction of the *ELMOD3* gene in patient-derived iPSCs with CRISPR/Cas9. (A) The schematic of gDNA targeting and mutation site G is highlighted in red. (B) Sanger sequencing confirmed that the *ELMOD3* mutation had been corrected successfully. The original heterozygous mutation was replaced by a new nonsense mutation. C(+/+): control iPSC. P(+/-): *ELMOD3*^*mut*^ iPSCs. P(+/+): *ELMOD3*^corrected^ iPSCs. (C) Sequencing results of 16 possible off-target sites.
(TIF)

S3 Fig qRT-PCR validation of the transcript levels of three group genes. Black bars indicate relative gene expression in *ELMOD3*^corrected^ iPSCs and gray bars in *ELMOD3*^mut^ iPSCs. All data are presented as the means±SD; the P-value was calculated by t-test. *: P < 0.05; **: P < 0.01; ****: P < 0.0001. (A-B) Intermediate filament cytoskeleton organization and mesenchyme migration group. (C-D) Ear morphogenesis group. (E-F) Potassium ion transport group.
(TIF)

S4 Fig Differentially expressed genes between *ELMOD3*^corrected^ and control iPSCs. (A) The heatmap showed a hierarchical clustering analysis of DEGs between *ELMOD3*^corrected^ and control iPSCs. Red and blue indicate genes with high and low expression levels, respectively. (B) Volcano plot showing the expression change of each gene and their significance. Red dots represent the expression of genes in *ELMOD3*^corrected^ iPSCs significantly up-regulated compared to normal control. Green dots represent the expression of genes in *ELMOD3*^corrected^ iPSCs significantly down-regulated compared to normal control. (C-D) Venn diagram representing the quantity of shared genes between group A (*ELMOD3*^mut^ vs control) and group B (*ELMOD3*^corrected^ vs control).
(TIF)

S1 Table The list of up-regulated genes between *ELMOD3*^mut^ and control iPSC lines. (XLSX)

S2 Table The list of down-regulated genes between *ELMOD3*^mut^ and control iPSC lines. (XLSX)

S3 Table 16 off-target sequences. (PDF)

S4 Table The list of up-regulated genes between *ELMOD3*^mut^ and *ELMOD3*^corrected^ iPSC lines. (XLSX)

S5 Table The list of down-regulated genes between *ELMOD3*^mut^ and *ELMOD3*^corrected^ iPSC lines. (XLSX)

S6 Table The list of up-regulated genes between *ELMOD3*^corrected^ and control iPSC lines. (XLSX)

S7 Table The list of down-regulated genes between *ELMOD3*^corrected^ and control iPSC lines. (XLSX)

S1 Raw images The original images. (TIF)
