# Supplementary figures and images for: Gene regulation analysis of patient-derived iPSCs and its CRISPR-corrected control provides a new tool for studying perturbations of ELMOD3 c.512A>G mutation during the development of inherited hearing loss
Source: PLoS One. 2023 Sep 14;18(9):e0288640. doi: 10.1371/journal.pone.0288640 (PMC10501637; doi:10.1371/journal.pone.0288640)

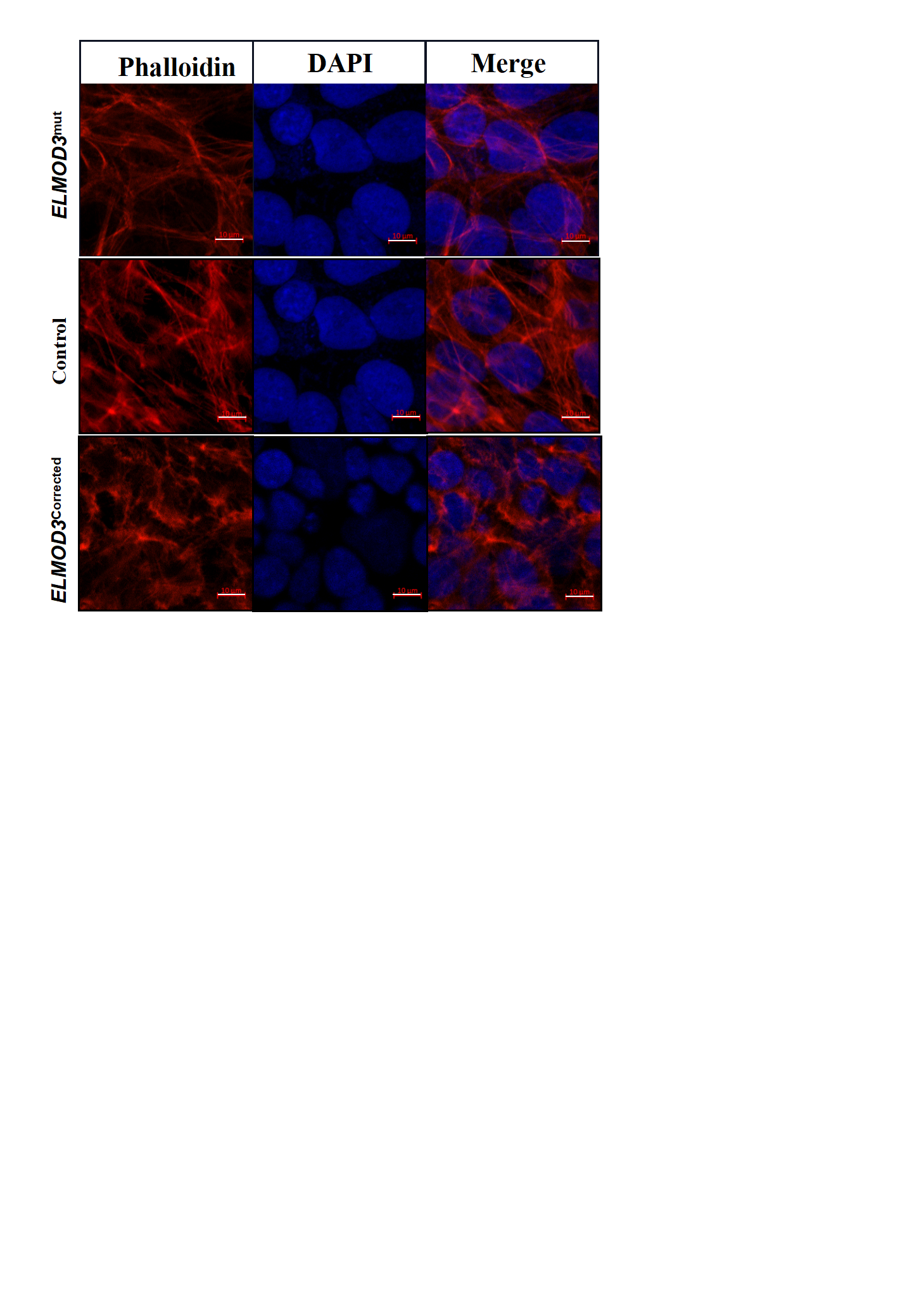

Supplement: S1 Fig — Bar, 100μM. (TIF) [file pone.0288640.s001.tif]

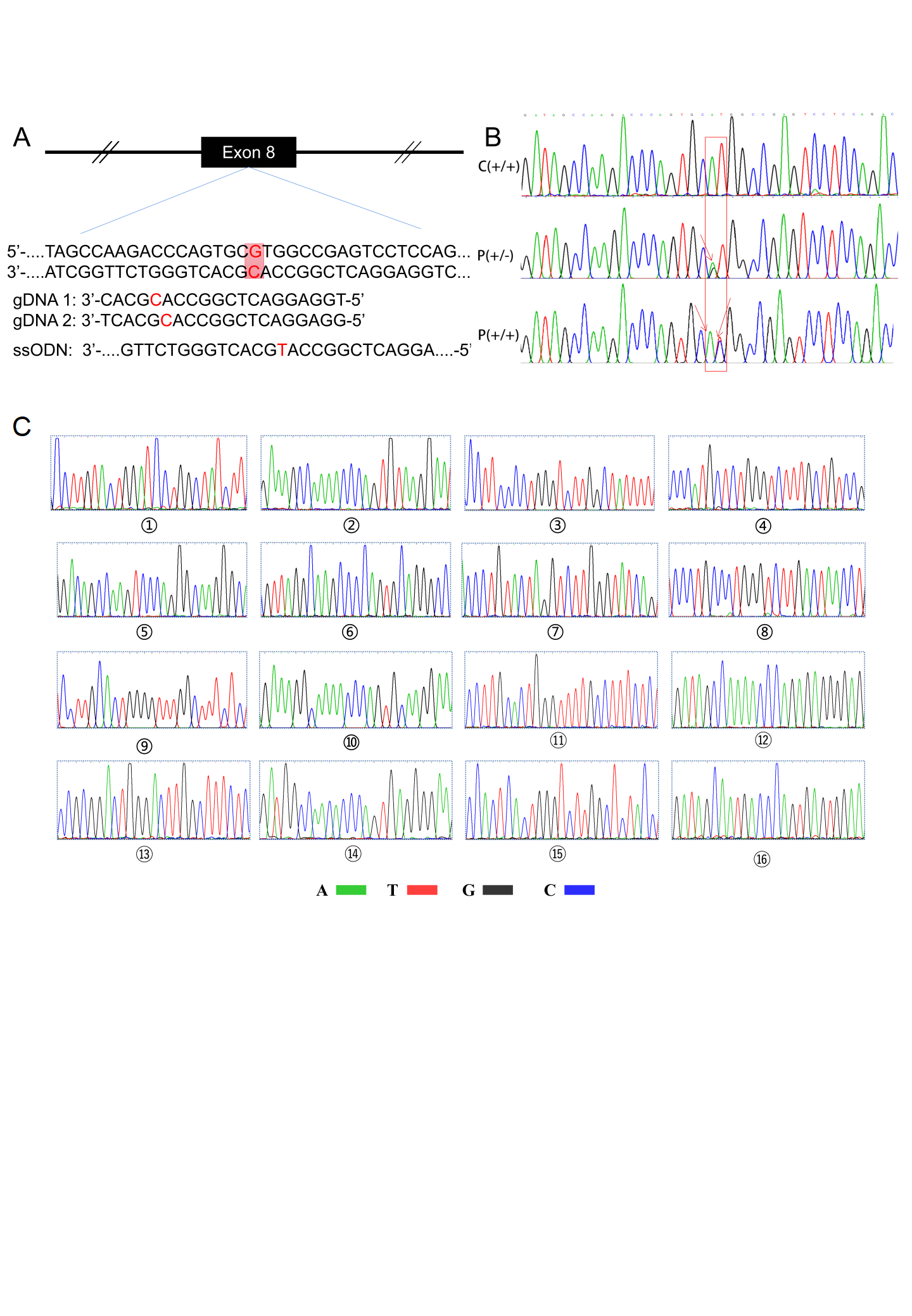

Supplement: S2 Fig — (A) The schematic of gDNA targeting and mutation site G is highlighted in red. (B) Sanger sequencing confirmed that the ELMOD3 mutation had been corrected successfully. The original heterozygous mutation was replaced by a new nonsense mutation. C(+/+): control iPSC. P(+/-): ELMOD3mut iPSCs. P(+/+): ELMOD3corrected iPSCs. (C) Sequencing results of 16 possible off-target sites. (TIF) [file pone.0288640.s002.tif]

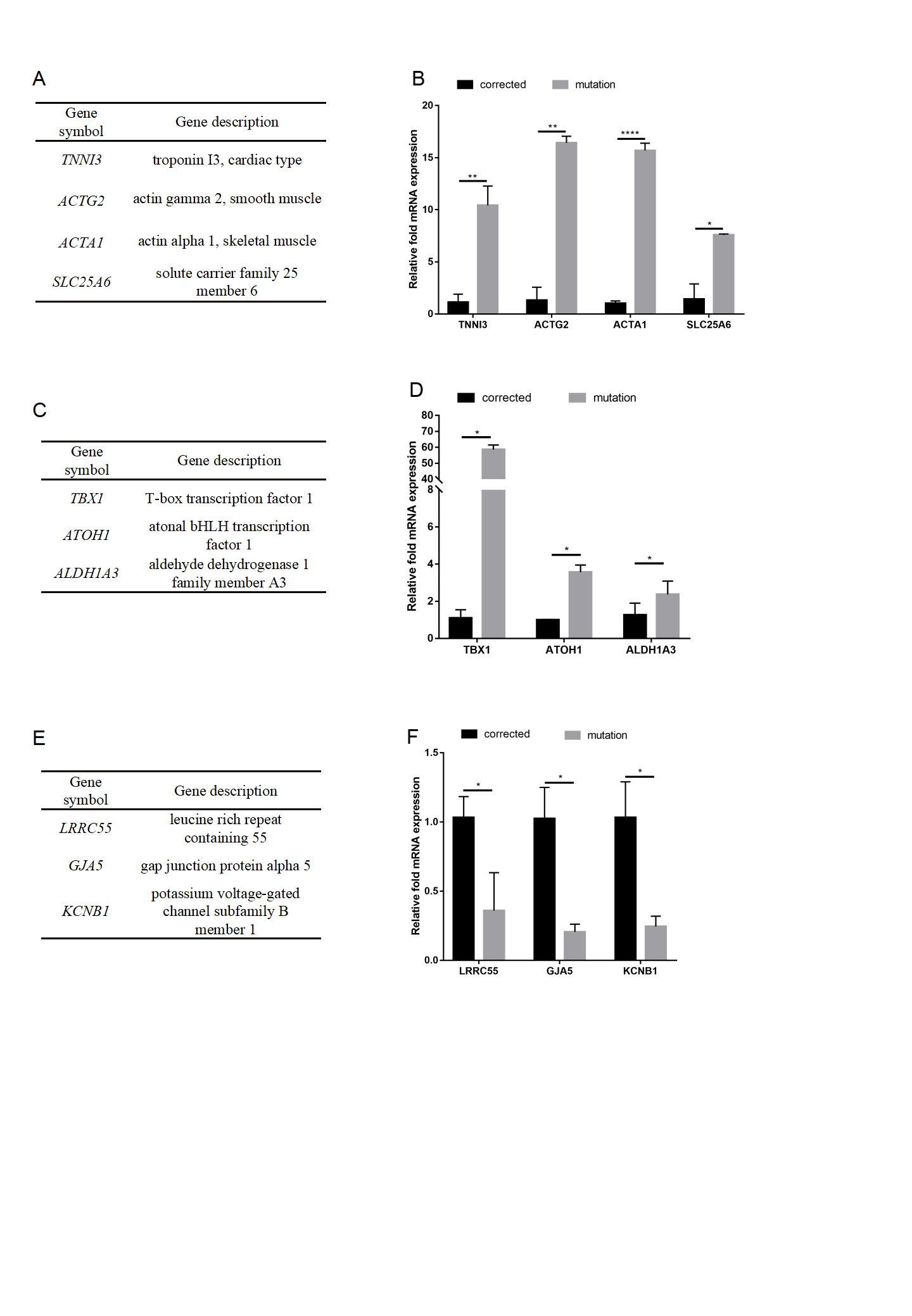

Supplement: S3 Fig — Black bars indicate relative gene expression in ELMOD3corrected iPSCs and gray bars in ELMOD3mut iPSCs. All data are presented as the means±SD; the P-value was calculated by t-test. *: P < 0.05; **: P < 0.01; ****: P < 0.0001. (A-B) Intermediate filament cytoskeleton organization and mesenchyme migration group. (C-D) Ear morphogenesis group. (E-F) Potassium ion transport group. (TIF) [file pone.0288640.s003.tif]

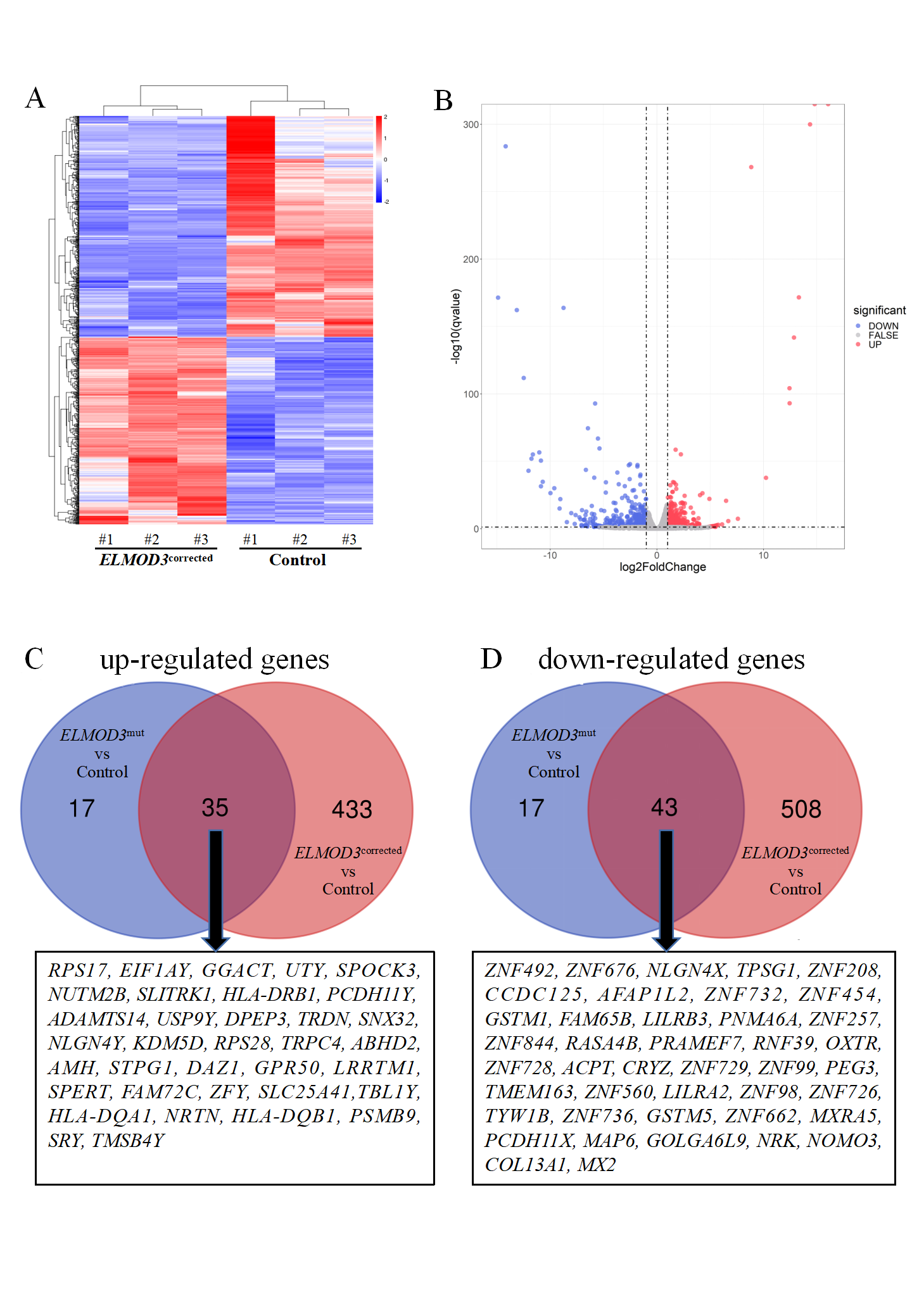

Supplement: S4 Fig — (A) The heatmap showed a hierarchical clustering analysis of DEGs between ELMOD3corrected and control iPSCs. Red and blue indicate genes with high and low expression levels, respectively. (B) Volcano plot showing the expression change of each gene and their significance. Red dots represent the expression of genes in ELMOD3corrected iPSCs significantly up-regulated compared to normal control. Green dots represent the expression of genes in ELMOD3corrected iPSCs significantly down-regulated compared to normal control. (C-D) Venn diagram representing the quantity of shared genes between group A (ELMOD3mut vs control) and group B (ELMOD3corrected vs control). (TIF) [file pone.0288640.s004.tif]

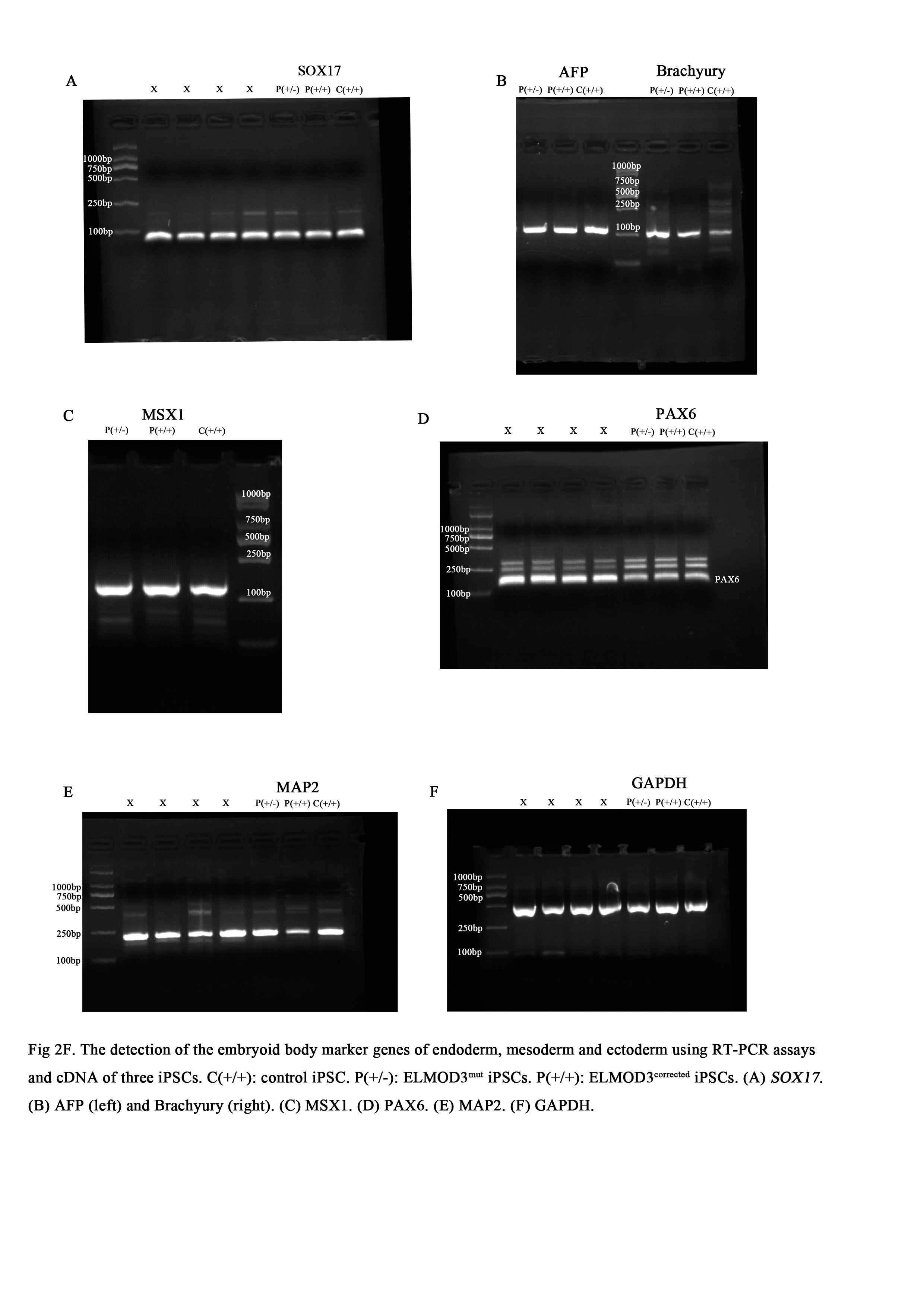

Supplement: S1 Raw images — (TIF) [file pone.0288640.s012.tif]
