# Supplementary material for: Gene regulation analysis of patient-derived iPSCs and its CRISPR-corrected control provides a new tool for studying perturbations of ELMOD3 c.512A>G mutation during the development of inherited hearing loss
Source: PLoS One. 2023 Sep 14;18(9):e0288640. doi: 10.1371/journal.pone.0288640 (PMC10501637; doi:10.1371/journal.pone.0288640)
Supplement: S3 Table — (PDF) [file pone.0288640.s007.pdf]

S3 Table 16 off-target sequences

Number	Location	Number of mismatches	Sequence (including mismatches)
1	chr1:76778989	3	CCTTGtACTGGaTCcTGGCTATC
2	chr12:72840610	3	GAaAGCCAAaACCCAGTGtAAGG
3	chr17:47279226	3	CCTTcCcCTGGGTCTTGGCTATt
4	chr19:48962683	3	CCATGCtCTGGGTCTTtGCTtTC
5	chr2:20145935	3	GAcAGCCAAGtCCCAGgGCAAGG
6	chr3:126518620	3	GtTAGgCAAGcCCCAGTGCAGGG
7	chr4:115610029	3	CCCTGtACTaGGTCTTGGgTATC
8	chr6:41504224	3	CCCTGCcCTGGGTCTTGaCTAaC
9	chr6:151647618	3	CCTTGCACTGGGgtTTGGCTtTC
10	chrX:83233585	3	GAaAGgCAAaACCCAGTGCAAGG
11	chrX:85759761	3	CCTTGCACTGGGTtTTGcCTtTC
12	chr12:24306685	3	GATAGCCAAaACCCAGaGaGGGG
13	chr16:1534223	3	CCGCGgACTGGGaCTTGGCTtTC
14	chr16:65626450	3	GATgGCCAAcACCCAGTGaGGGG
15	chr3:184456679	3	CCACtCACTGGGTCTTGGCTcTa
16	chr8:19249682	3	GATAGCaAtGACCCAGTGaGTGG
